# Three-Year Corneal Biomechanical Outcomes Assessed by CORVIS ST Dynamic Scheimpflug Imaging After Epithelium-Off Conventional Versus Accelerated Cross-Linking in Progressive Keratoconus

**DOI:** 10.22336/rjo.2026.13

**Published:** 2026

**Authors:** Radu Nicolae Pop, Cristina Nicula, Patricia Nicula, Dorin Nicula, Bianca Pop

**Affiliations:** 1 OCULENS Ophthalmology Clinic, Cluj-Napoca, Romania; 2 “Lucian Blaga” University, Faculty of Medicine, Sibiu, Romania; 3 Department of Oral and Maxillofacial Surgery and Radiology, “Iuliu Hațieganu” University of Medicine and Pharmacy, Faculty of Dentistry, Cluj-Napoca, Romania; 4 Surgical Department, University of Agricultural Sciences and Veterinary Medicine, Cluj-Napoca, Romania

**Keywords:** corneal cross-linking, keratoconus, Corvis ST, biomechanics, stiffness, deformation amplitude, BAD-D, conventional CXL, accelerated CXL, KCN = Keratoconus, CXL = Cross-linking, C-CXL = Conventional cross-linking, A-CXL = Accelerated cross-linking, UVA = Ultraviolet A, UCVA = Uncorrected visual acuity, CDVA = Corrected distance visual acuity, Kmax = Maximum keratometry, D = Diopter, DA = Deformation amplitude, A1T, A2T = First and second applanation times, PD = Peak distance, HC radius = Highest concavity radius, SP-A1 = Stiffness parameter at first applanation, BAD-D = Belin/Ambrosio Enhanced Ectasia Total Deviation Index, APLANO-T = Applanation tonometry, bIOP = Biomechanically corrected intraocular pressure, CBI = Corvis biomechanical index, DCR = Dynamic corneal response, CCT = Central corneal thickness

## Abstract

**Purpose:**

To compare long-term corneal biomechanical and tomographic responses using dynamic Scheimpflug analysis, three years after epithelium-off (epi-off) conventional corneal collagen cross-linking (C-CXL; Dresden protocol) versus epi-off accelerated cross-linking (A-CXL) in patients with progressive keratoconus.

**Methods:**

Prospective comparative study. 81 patients were assessed; treatment was performed in 40, including 23 bilateral keratoconus treatments and 17 unilateral treatments, resulting in 63 eyes included in the final 36-month analysis cohort. Eyes underwent epi-off CXL with either conventional protocol (3 mW/cm^2^ for 30 min; 5.4 J/cm^2^; n=34) or accelerated protocol (9 mW/cm^2^ for 10 min; 5.4 J/cm^2^; n=29). Assessments at baseline, 6, 12, 24, and 36 months included Kmax, uncorrected visual acuity (UCVA), corrected distance visual acuity (CDVA), pachymetry, and Corvis ST dynamic corneal response parameters: deformation amplitude (DA), applanation times (A1T, A2T), peak distance (PD), highest concavity (HC) radius, stiffness parameter at first applanation (SP-A1), and BAD-D. Primary endpoint: between-group differences at 36 months and longitudinal change in key biomechanical parameters—secondary endpoints: Kmax, UCVA, and CDVA at each follow-up. Welch’s t-test compared groups at each time point; repeated-measures modeling evaluated time, group, and time × group interaction.

**Results:**

Both protocols demonstrated significant longitudinal biomechanical remodeling and clinical stabilization over 36 months (time effect p < 0.001 for DA, SP-A1, A1T, A2T, PD, and HC radius). At 36 months, between-group differences were not statistically significant for Kmax, UCVA, CDVA, pachymetry, DA, A1T, A2T, PD, HC radius, SP-A1, or BAD-D (all p > 0.05). Longitudinal modeling demonstrated a significant time × group interaction for SP-A1 (p=0.042), indicating a modest protocol-dependent divergence in stiffness trajectory over time, while DA, A1T/A2T, PD, and HC radius showed parallel improvements without significant interaction. Pachymetry showed early thinning (6 months) followed by remodeling and stability by 36 months. No sight-threatening complications occurred.

**Discussions:**

Both epi-off conventional and accelerated CXL produced sustained biomechanical remodeling and clinical stabilization over 36 months. Although final between-group differences were not statistically significant for Kmax, visual acuity, pachymetry, deformation amplitude, applanation times, peak distance, HC radius, SP-A1, or BAD-D, repeated-measures analysis identified a significant time × group interaction for SP-A1, suggesting a modest protocol-dependent divergence in the trajectory of corneal stiffening. These findings indicate that matching total fluence does not necessarily produce identical biomechanical time courses and that irradiation duration may still influence the pattern of postoperative strengthening.

**Conclusions:**

Three years after treatment, both epi-off C-CXL and epi-off A-CXL stabilized progressive keratoconus and produced sustained biomechanical remodeling detectable by dynamic Scheimpflug imaging. Final 36-month outcomes were comparable between protocols in this cohort; however, the stiffness trajectory (SP-A1) differed modestly over time, supporting the concept that irradiation duration may influence the time-course of biomechanical strengthening even when total fluence is matched.

## Introduction

Keratoconus (KCN) is a progressive, noninflammatory ectatic disease characterized by stromal weakening and thinning, biomechanical instability, and corneal deformation, leading to irregular astigmatism and visual impairment. The disease’s hallmark is biomechanical failure, not simply topographic steepening. Therefore, evaluating true corneal stiffness is crucial.

From an epidemiological perspective, modern diagnostics and registries suggest a higher burden than historically reported. A recent global systematic review and meta-analysis estimated a pooled worldwide prevalence of approximately 289 per 100,000 persons (≈0.24%) and an incidence of ~4.0 per 100,000 person-years, with peak rates in young adults (20-29 years) [1]. European registry-based data demonstrate notable variability, reflecting differences in case ascertainment, ethnic distribution, screening intensity, and health system reporting. For example, Denmark reported an incidence of approximately 3.6 per 100,000 person-years in the modern era of registry completeness [2], while Swedish nationwide registry data suggested an annual incidence of ~11.8 per 100,000 with the highest incidence in the third decade of life [3]. These epidemiologic data emphasize that keratoconus is relatively common among young patients and remains a major driver of ectasia stabilization strategies.

**Fig. 1:**
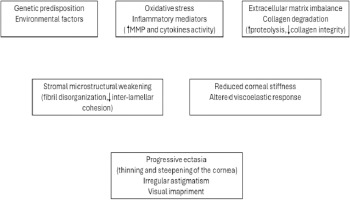
Keratoconus physiopathology

Regarding physiopathology, keratoconus is increasingly recognized as a disease characterized by biomechanical failure due to altered stromal microstructure and biochemical dysregulation, rather than a purely geometric disorder. Histopathology commonly shows stromal thinning, fragmentation/breaks in Bowman’s layer, epithelial thinning, and iron deposition in the basal epithelium, alongside focal scarring in more advanced disease [4]. Contemporary reviews also support a role for inflammatory and oxidative stress pathways, altered extracellular matrix turnover, and keratocyte dysfunction, which together contribute to stromal weakening and progressive ectasia (**Fig. 1**) [5].

**Fig. 2:**
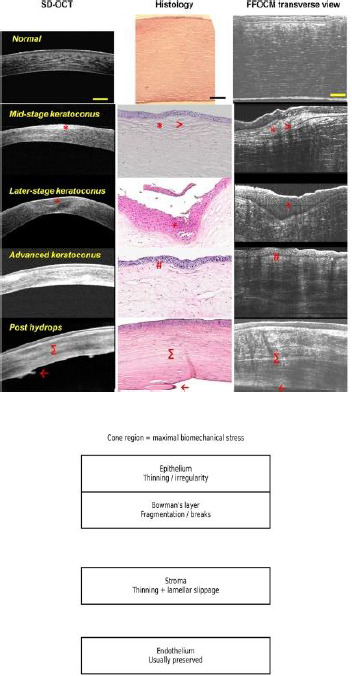
Keratoconus histology

From a histopathology standpoint, epi-off CXL produces a characteristic sequence of tissue-level changes. Early after treatment, keratocyte apoptosis in the anterior stroma is followed by repopulation and long-term extracellular matrix remodeling. The well-described stromal demarcation line marks the transition between cross-linked anterior stroma and untreated posterior stroma and is considered a clinical marker of treatment-effect depth; its depth varies with protocol and oxygen kinetics [6]. Histopathologic studies of corneas that later required keratoplasty after prior CXL have reported persistent structural and cellular alterations consistent with long-term remodeling rather than transient changes (**Fig. 2**) [7].

**Fig. 3:**
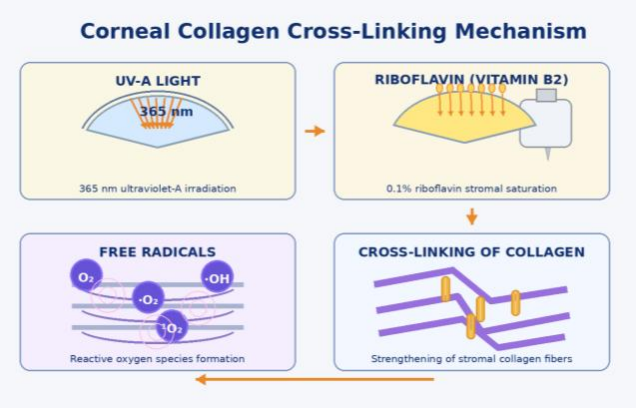
Mechanistic overview of corneal collagen cross-linking Source: Original drawn by the authors, based on published descriptions of riboflavin/UVA photo-oxidative cross-linking mechanisms and protocol schematics [8-17]

Corneal collagen cross-linking (CXL), using riboflavin and ultraviolet-A (UVA), remains the only widely adopted therapy to halt progression and biomechanically stabilize the cornea, thereby preventing keratoplasty. The treatment modifies stromal biomechanics by inducing covalent bonds between collagen fibrils via riboflavin-mediated photo-oxidative reactions (**Fig. 3**) [8,9].

Conventional protocols provide extensive cross-link formation but are time-consuming. Accelerated CXL, which uses higher irradiance for a shorter duration, is based on the Bunsen-Roscoe reciprocity law and was introduced to shorten treatment time while preserving biomechanical efficacy. However, full biomechanical equivalence may not apply in living corneal tissue because oxygen diffusion and photochemical kinetics can limit the reciprocity effect [10-17].

**Fig. 4:**
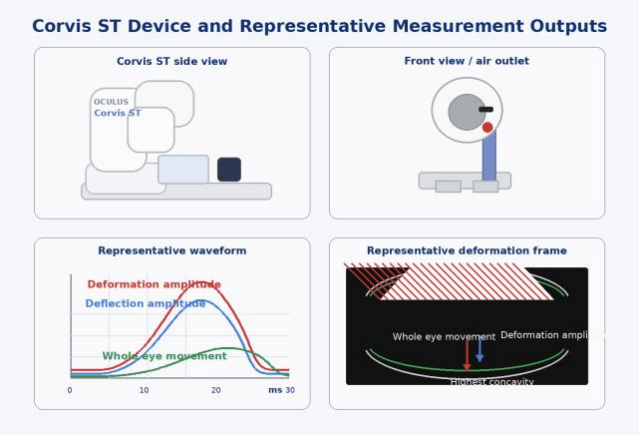
Corvis ST device and representative measurement outputs Source: Original drawn by the authors, based on published descriptions of the Corvis ST platform, air-puff measurement principle, and dynamic Scheimpflug outputs [18-20]

Dynamic Scheimpflug imaging allows in vivo quantification of corneal viscoelastic behavior under a calibrated air impulse. The Corvis ST (Oculus, Wetzlar, Germany) (**Fig. 4**) is a non-contact tonometer combined with a high-speed corneal biomechanics analyzer. It delivers an air puff and records more than 4,300 Scheimpflug frames per second during corneal deformation, enabling in vivo biomechanical analysis [18-20].

Intraocular Pressure (IOP) - IOP (non-corrected) - Goldmann-correlated pressure estimate and bIOP (biomechanically corrected IOP), were adjusted for corneal stiffness & thickness (more accurate in post-LASIK/keratoconus eyes).

**Fig. 5:**
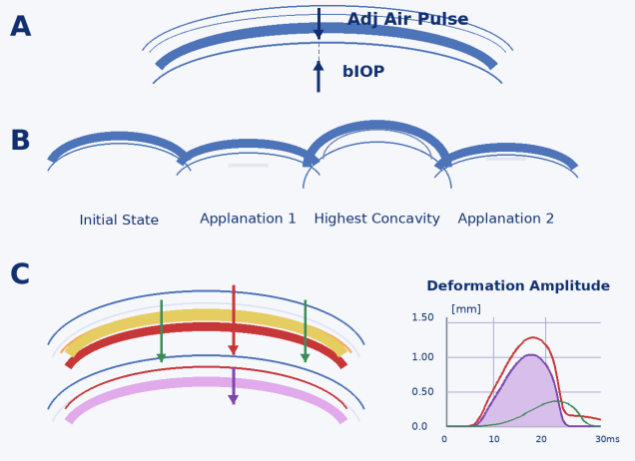
Corvis ST air-puff phases, bIOP context, and deformation amplitude components Source: Original drawn by the authors, based on published descriptions of Corvis ST air-puff phases, biomechanically corrected IOP, deformation amplitude, and dynamic corneal response parameters [18-20]

From that dynamic response, it extracts three major categories of parameters:

a) achymetry & Geometry: Central Corneal Thickness (CCT); Pachymetry at apex *;* Corneal curvature at highest concavity *;* Corneal radius (dynamic) **(Fig. 5**);

IOP and corneal geometry are essential contextual variables because they influence biomechanical responses.

**Fig. 6:**
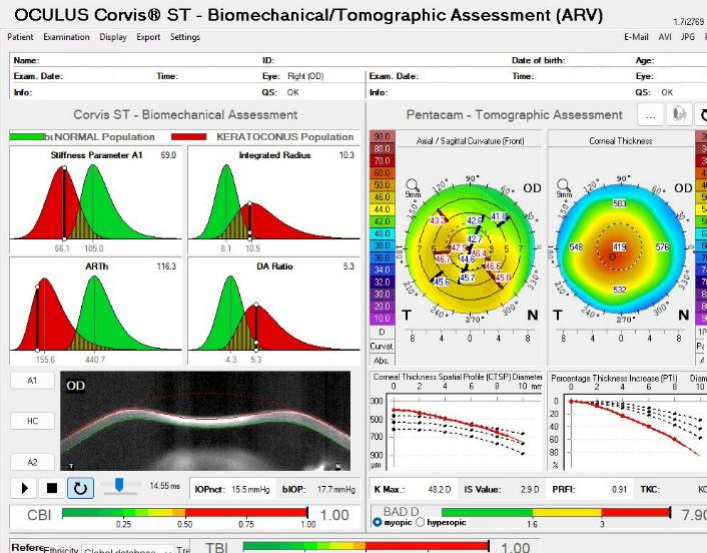
Corvis ST mechanism of measurement

b) Dynamic Corneal Response (DCR) parameters and the highest concavity phase (**Fig. 6**);

These describe how the cornea moves during the air puff. The main deformation events captured by CORVIS ST are, by sequence:

Pre-Air Puff → A1 (First Applanation) → HC (Highest Concavity) → A2 (Second Applanation) → Recoil

Applanation Phase Metrics are the following: A1 time - time to first flattening; A1 velocity - speed of cornea moving inward; A1 length - flattened corneal length; A2 time - time to second flattening; A2 velocity - speed of cornea moving outward; A2 length - flattened length during return.

These quantify the temporal and spatial deformation responses during and after the two air-puff-induced applanation events [18-20].

Highest concavity (HC) metrics: Deformation Amplitude (DA) - maximum central displacement of cornea (mm); Peak Distance (PD) - distance between two highest bending “peaks” at HC (mm); Radius Curvature (RC) at maximum concavity (mm); Time to reach HC (ms).

HC parameters reflect how far and in what geometric pattern the cornea deforms under a fixed load, and they are commonly associated with biomechanical stiffness [18-20].

c) Integrated Biomechanical Indices

**Fig. 7:**
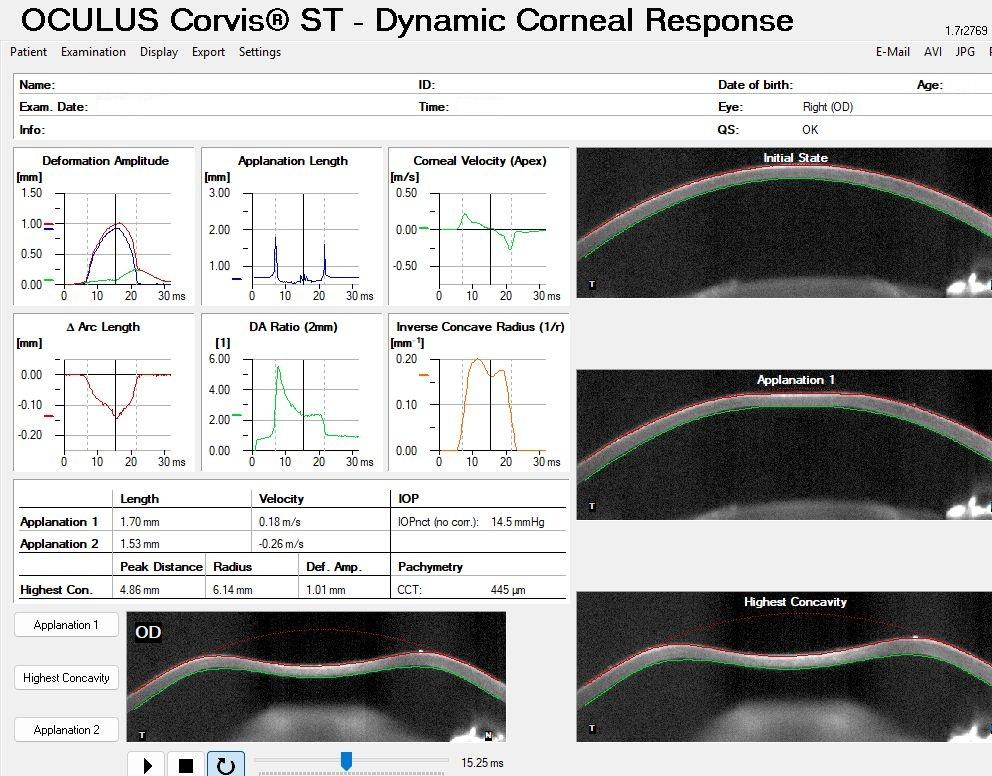
Corvis ST parameters

These integrate multiple DCR metrics to estimate tissue behavior and disease risk (**Fig. 7**):

Stiffness Parameter at first applanation - global stiffness measure (SP-A1); Stiffness Parameter at first applanation - global stiffness measure (DA ratio max 2 mm); Belin-Ambrósio Deviation Index (BAD-D) comes from OCULUS Optikgeräte GmbH Pentacam tomography, but it is clinically paired with CORVIS indices - to asses combined anterior and posterior elevation, pachymetry progression, and relational thickness deviation from normal; Corvis Biomechanical Index (CBI) - composite index for detecting ectatic corneas; Tomographic Biomechanical Index (TBI) - AI-based combination of BAD-D (tomography) + CBI (biomechanics) with high sensitivity in early ectasia detection.

Indices such as TBI and CBI are widely used to improve the detection of early keratoconus and other ectatic diseases [21].

Long-term comparative data using biomechanical endpoints remain limited. This study addresses that gap by evaluating 3-year outcomes after epi-off conventional versus accelerated CXL in a cohort of 63 eyes (34 conventional, 29 accelerated) to (1) quantify and compare biomechanical remodeling over 3 years, (2) evaluate K-max and visual acuity outcomes, (3) evaluate CORVIS ST biomechanical parameters, and (4) place the results in context with recent literature.

## Material and methods

### Study design and participants

This was a prospective, interventional, comparative study with longitudinal follow-up. Eighty-one patients were assessed. Treatment was performed in 40 patients, including 23 bilateral keratoconus treatments and 17 unilateral treatments, resulting in 63 treated eyes included in the final analysis cohort. All cases were treated at OCULENS Ophthalmology Clinic (Cluj-Napoca, Romania) between 2019 and 2022, with follow-up through 2025.

The final cohort included 34 eyes treated with C-CXL and 29 eyes treated with A-CXL.

### Inclusion criteria

Eyes were eligible if all the following were met:

- Disease and progression criteria
  Clinical diagnosis of keratoconus, stages I to III, according to the Amsler-Krumeich classification, based on refracto-keratometry, corneal tomography, and slit-lamp findings, including at least one of: Asymmetric inferior-superior steepening; Skewed radial axis; Posterior elevation abnormalities; Stromal thinning with or without Fleischer ring or Vogt striae.
  Documented refracto-keratometrical and tomographical progression within 12 months, defined by ≥ 1 of: Increase in K-max ≥ 1.0 D; Increase in manifest cylinder ≥ 1.0 D; Reduction in minimal pachymetry ≥ 10 μm; Deterioration of CDVA ≥ 0.1 logMAR not attributable to other pathology.
  K-max < 65.0 D (to avoid extremely advanced ectasia with poor visual potential).
- Anatomical and safety criteria
  Minimal stromal thickness ≥ 400 μm after epithelial removal and riboflavin saturation; Clear central cornea without visually significant scarring; Endothelial cell density measured by specular microscopy, within age-appropriate normal range; Absence of active ocular surface disease (Severe dry eye, SICCA Keratoconjunctivitis, Sjogren Syndrome, …).
- General criteria
  Age ≥ 17 years (skeletally mature corneas); Ability to comply with follow-up visits up to 36 months; No previous CXL in the study eye.

### Exclusion criteria

Eyes were excluded if any of the following were present:

Ocular Exclusions - Prior corneal surgery (e.g., keratoplasty, refractive surgery, intracorneal ring); Central corneal scarring or opacification affecting visual axis; Hydrops or history of acute corneal hydrops; Severe dry eye or ocular surface disease impairing epithelial healing; Active ocular infection or inflammation; Herpetic keratitis history; Autoimmune or connective tissue disorders affecting corneal healing; Endothelial dysfunction or Fuchs dystrophy; Intraocular pressure > 21 mmHg or glaucoma with optic nerve damage; Pregnancy-related corneal changes (temporary keratoconus progression).

Systemic Exclusions - Pregnancy or breastfeeding at time of procedure; Systemic collagen vascular disease; Immunosuppressive therapy; Diabetes with poor wound healing; History of keloid formation or abnormal scarring response.

### Protocol exclusions

Cases were excluded if: corneal thickness < 400 μm after riboflavin soak; Inability to complete UVA exposure safely; Non-compliance with postoperative medication regimen.

### Ethical approval and patient consent

The study adhered to the tenets of the Declaration of Helsinki and to institutional regulations governing human subjects research, as per the clinic’s ethical committee, number 1/2026.

All patients provided written informed consent before undergoing cross-linking. The consent process included detailed discussion of: Nature and purpose of CXL treatment; Differences between conventional and accelerated protocols; Treatment benefits (disease stabilization) to halt progression and prevent keratoplasty; Possible treatment risks and complications (pain, infection, haze, scarring, delayed epithelial healing, rare endothelial damage); Long term need of glasses or rigid/hybrid contact lenses; Alternative options where applicable (intracorneal ring segments, topography guided photorefractive keratectomy combined with CXL); Possibility of continuous progression and requiring additional or re-treatment.

Patients were informed that biomechanical imaging data (CORVIS ST) and tomography would be used for research analysis in anonymized form.

Consent also covered long-term follow-up imaging and data publication in scientific journals without identifying information.

Ocular examination before treatment included: Visual acuity (VA), uncorrected (UCVA) and corrected distance (CDVA), measured in logMAR scale; Refractometry and keratometry; Slit lamp examination; Intra-ocular pressure (APLANO-T and bIOP); Endothelial cell count (specular microscopy); Corneal tomography (including pachymetry), PENTACAM and biomechanical CORVIS ST parameters.

CORVIS ST (dynamic Scheimpflug) measurements were obtained at each visit per manufacturer recommendations. Parameters examined included: Deformation amplitude (DA, mm) measures maximal apical displacement; First applanation time (A1T, ms) and second applanation time (A2T, ms); Applanation velocities (V1, V2) - derived from applanation time and displacement (reported here implicitly by reporting A1T and A2T and the SP-A1 parameter); Peak distance (PD) and HC radius (mm) - geometry at highest concavity; Stiffness parameter at first applanation (SP-A1) - estimate stiffness at first applanation; Highest concavity (HC) radius (mm) - it measures the curvature radius at the corneal apex during the highest concavity phase; Belin/Ambrosio Enhanced Ectasia Total Deviation Index (BAD-D) - tomographic measurement from the PENTACAM that analyzes corneal thickness and elevation.

Measurements were collected at baseline (preoperatively) and at 6, 12, 24, and 36 months.

**Fig. 8:**
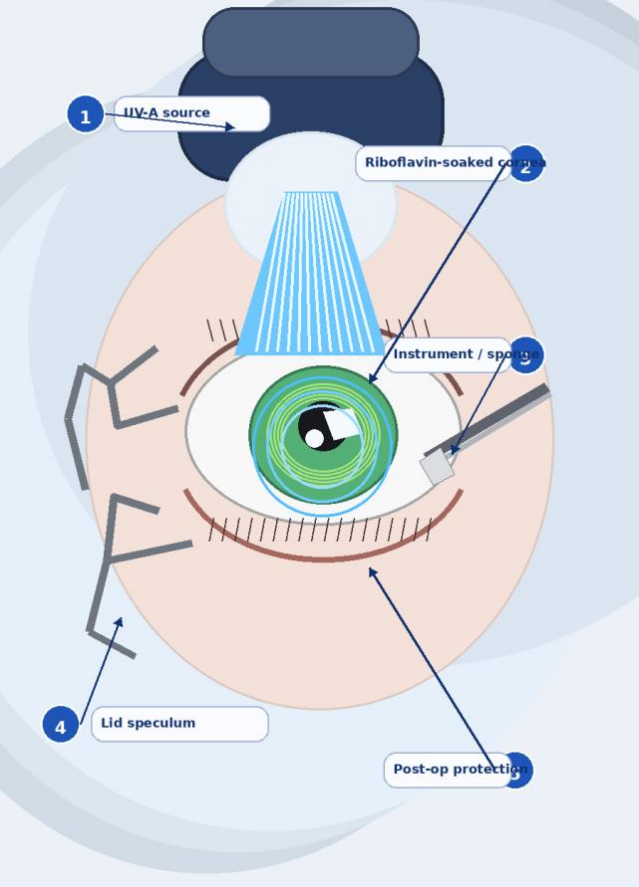
Illustrative clinical view of epithelium-off corneal collagen cross-linking Source: Original drawn by the authors, based on standard epithelium-off corneal collagen cross-linking procedural steps described in references [8,13-17]

The cross-linking technique was performed as follows (**Fig. 8**): Opening of the riboflavin 0,1%-dextran 20% solution under sterile conditions in the operating room. After verifying the power of the UVA illuminator, topical anesthesia (Alcaine solution) was applied in 3-4 drops 15-20 minutes before cross-linking. Corneal de-epithelization was manually performed on a ~ 9 mm diameter. Instillation of 0,1% riboflavin every 3 minutes for 30 minutes before irradiation, as a corneal soak. Exposure of the central part of the cornea to UVA light and instillation of riboflavin were performed for CCXL: every 3 minutes for 30 minutes, and for ACXL: every 2 minutes for 10 minutes. After treatment finalization and instillation of antibiotics and steroids, a therapeutic contact lens was applied (for 2-3 days), and an eye patch was applied (removed after 24 hours).

### Statistical Analysis

Descriptive statistics were presented as mean ± standard deviation. Between-group comparisons at each follow-up were conducted using independent-samples t-tests (Welch’s t-test when variances were unequal). Longitudinal summaries (means ± SEM) are presented in figures. Statistical significance was set at p < 0.05. Normality of distributions was verified using the Shapiro-Wilk test, and homogeneity of variances was assessed using Levene’s test.

### Rationale for the statistical model

Because each eye was measured repeatedly over **four postoperative time points (6, 12, 24, 36 months)**, observations were **not independent**. Therefore, a **repeated-measures ANOVA (RM-ANOVA)** model was appropriate to evaluate:

1. **Time effect** → Does the parameter change after CXL regardless of protocol?
2. **Group effect** → Are overall values different between conventional and accelerated CXL?
3. **Time × Group interaction** → Do the *patterns of change over time* differ between protocols?

This interaction term is the **most clinically relevant** because it indicates whether one protocol produces a different *trajectory* of biomechanical recovery.

## Results

1. Baseline characteristics

Sixty-three eyes were included in the final analysis cohort (34 C-CXL; 29 A-CXL). Baseline demographic and biomechanical characteristics were comparable between groups. There were no statistically significant baseline differences in age (p=0.12), Kmax (p=0.71), UCVA (p=0.75), CDVA (p=0.45), DA (p=0.71), SP-A1 (p=0.68), BAD-D (p=0.26), A1T (p=0.73), A2T (p=0.80), PD (p=0.82), or HC radius (p=0.66) (**Table 1**).

**Table 1:** Baseline characteristics (Welch’s t-test)

Variable	C-CXL Mean ± SD	A-CXL Mean ± SD	p (Welch t)
Age	25.51 ± 4.69	27.49 ± 5.17	0.1202
Kmax	58.17 ± 4.06	57.79 ± 3.97	0.7082
Pachymetry	468.47 ± 19.93	462.09 ± 21.71	0.2323
Deformation_Amplitude	1.15 ± 0.08	1.14 ± 0.10	0.7052
SP_A1	70.77 ± 6.37	70.05 ± 7.17	0.6775
HC_Radius	7.49 ± 0.61	7.56 ± 0.55	0.6618
BAD_D	5.40 ± 1.02	5.08 ± 1.13	0.2567
UCVA_logMAR	0.62 ± 0.17	0.60 ± 0.16	0.7174
CDVA_logMAR	0.25 ± 0.09	0.23 ± 0.08	0.4298

2. Keratometric and pachymetric outcomes

**Fig. 9:**
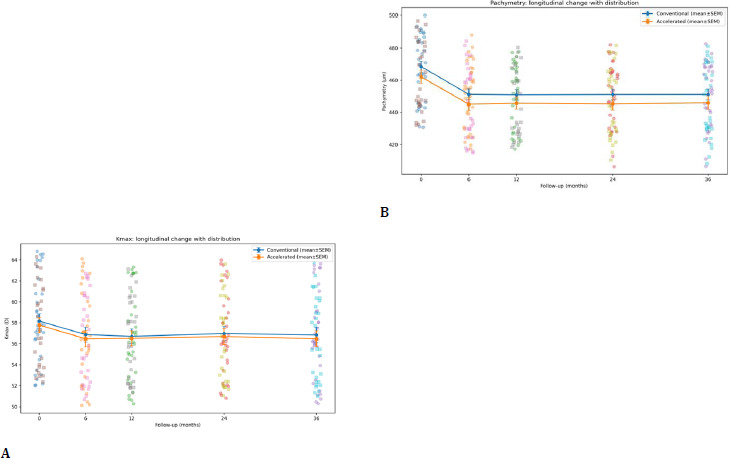
A, B Mean Kmax and pachymetry changes over 36 months in conventional versus accelerated CXL groups

Both protocols resulted in longitudinal stabilization and remodeling of Kmax and pachymetry over 36 months (time effect p < 0.001). At 36 months, between-group differences were not statistically significant for Kmax (p=0.73). Pachymetry demonstrated the typical postoperative profile, characterized by early thinning at 6 months, followed by remodeling between 6 and 24 months and stability at 36 months. At 36 months, pachymetry did not differ significantly between protocols (p=0.35) (**Fig. 9A, B, Table 2, 3**).

**Table 2:** Kmax (D) over time (mean ± SD) and between-group p-values

Group	Baseline	6m	12m	24m	36m
C-CXL	58.17 ± 4.06	56.88 ± 4.17	56.71 ± 4.10	56.97 ± 3.97	56.84 ± 4.18
A-CXL	57.79 ± 3.97	56.48 ± 4.16	56.52 ± 3.87	56.69 ± 4.11	56.48 ± 4.01
p (Welch)	0.708	0.702	0.853	0.783	0.730

**Table 3:** Pachymetry (µm) over time (mean ± SD) and between-group p-values

Group	Baseline	6m	12m	24m	36m
C-CXL	468.47 ± 19.93	451.24 ± 20.02	450.96 ± 19.24	451.12 ± 19.47	451.14 ± 20.31
A-CXL	462.09 ± 21.71	445.08 ± 22.64	445.71 ± 20.55	445.29 ± 22.03	445.98 ± 22.67
p (Welch)	0.232	0.261	0.303	0.274	0.349

3. Visual acuity outcomes

**Fig. 10:**
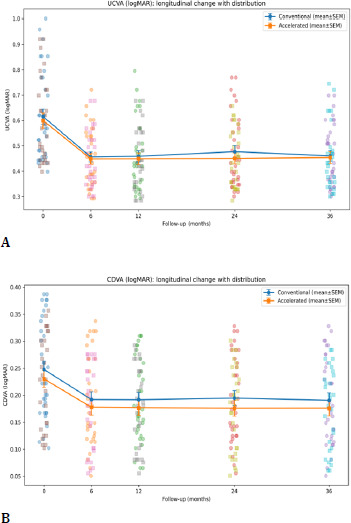
UCVA (**A**); CDVA (**B**)

UCVA and CDVA demonstrated significant longitudinal change after CXL over 36 months (time effect p < 0.001). At 36 months, UCVA and CDVA did not differ significantly between groups (UCVA p=0.82; CDVA p=0.47). Visual improvements occurred gradually, with the most pronounced changes typically observed during the first 12-24 months, consistent with the phase of biomechanical maturation and corneal remodeling. No eye experienced sustained loss of ≥ 2 lines of corrected vision (**Table 4, 5, Fig. 10A, B**).

**Table 4:** UCVA (logMAR) over time (mean ± SD) and between-group p-values

Group	Baseline	6m	12m	24m	36m
C-CXL	0.62 ± 0.17	0.46 ± 0.11	0.46 ± 0.13	0.48 ± 0.14	0.46 ± 0.12
A-CXL	0.60 ± 0.16	0.45 ± 0.11	0.45 ± 0.12	0.45 ± 0.11	0.45 ± 0.12
p (Welch)	0.717	0.790	0.773	0.412	0.839

**Table 5:** CDVA (logMAR) over time (mean ± SD) and between-group p-values

Group	Baseline	6m	12m	24m	36m
C-CXL	0.25 ± 0.09	0.19 ± 0.08	0.19 ± 0.08	0.19 ± 0.08	0.19 ± 0.08
A-CXL	0.23 ± 0.08	0.18 ± 0.08	0.18 ± 0.08	0.18 ± 0.08	0.18 ± 0.07
p (Welch)	0.430	0.487	0.441	0.339	0.450

4. CORVIS ST biomechanical changes

Dynamic Scheimpflug analysis revealed significant and sustained biomechanical changes following CXL in both groups, with a coherent pattern across deformation, timing, and concavity geometry.

Deformation amplitude (DA) decreased significantly over time (time effect p < 0.001), reflecting reduced corneal deformability under standardized air-puff loading. Applanation timing parameters (A1T and A2T) lengthened significantly over time (both time effects p < 0.001), consistent with increased resistance to deformation and delayed recovery. At the highest concavity, peak distance (PD) decreased, and HC radius increased significantly over time (time effects p < 0.001), indicating reduced corneal bending and altered concavity geometry consistent with stiffening.

**Fig. 11:**
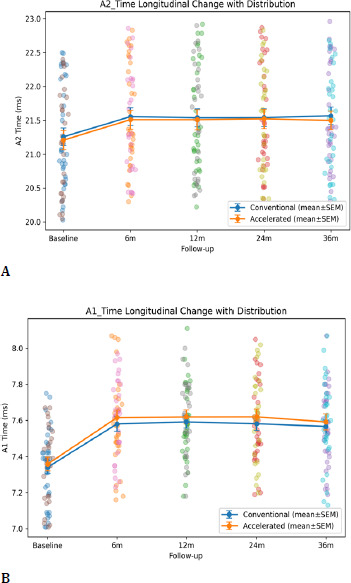
Deformation amplitude (A); Deformation amplitude (B)

**Fig. 12:**
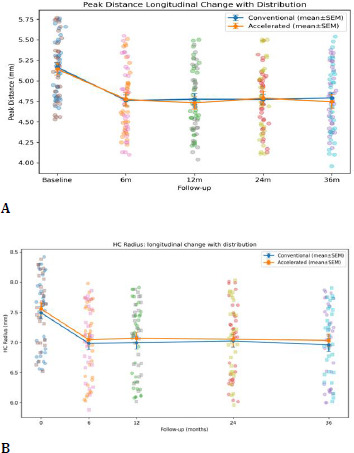
A1T and A2T

**Fig. 13:**
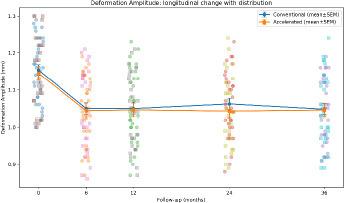
Peak distance (A); HC radius (B)

**Fig. 14:**
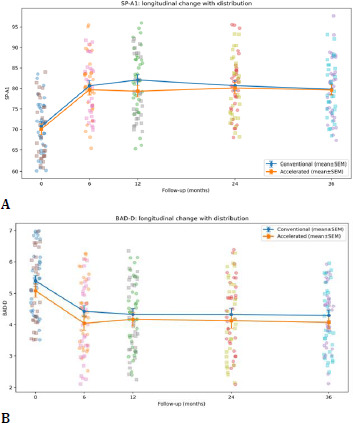
SP-A1 (A); BAD-D (B)

SP-A1 increased significantly over time (time effect p < 0.001), confirming increased stiffness at first applanation. At 36 months, between-group differences were not statistically significant for DA (p=0.92), A1T (p=0.66), A2T (p=0.72), PD (p=0.67), HC radius (p=0.62), SP-A1 (p=0.92), or BAD-D (p=0.40). However, repeated-measures modeling demonstrated a significant time × group interaction for SP-A1 (p=0.042), indicating a modest protocol-dependent divergence in stiffness trajectory over time. In contrast, DA, A1T/A2T, PD, and HC radius did not show significant interaction (p > 0.05) (**Table 6, Fig. 11A, B, Fig. 12, Fig. 13A, B, Fig. 14A, B**).

**Table 6:** Visual and Biomechanical Outcomes After CXL (Combined Table)

Parameter	Group	Baseline	6m	12m	24m	36m	p (36m) / η^2^
CDVA (logMAR)	C-CXL	0.25 ± 0.09	0.19 ± 0.08	0.19 ± 0.08	0.19 ± 0.08	0.19 ± 0.08	0.450 / 0.12
	A-CXL	0.23 ± 0.08	0.18 ± 0.08	0.18 ± 0.08	0.18 ± 0.08	0.18 ± 0.07	
Deformation Amplitude (mm)	C-CXL	1.15 ± 0.08	1.05 ± 0.09	1.05 ± 0.10	1.06 ± 0.09	1.05 ± 0.09	0.925 / 0.41
	A-CXL	1.14 ± 0.10	1.04 ± 0.10	1.05 ± 0.10	1.04 ± 0.10	1.04 ± 0.10	
SP-A1	C-CXL	70.77±6.37	80.63±7.22	82.08±7.81	80.66±7.06	79.83±7.43	0.916 / 0.49
	A-CXL	70.05 ± 7.17	79.70 ± 7.18	79.35 ± 7.53	80.10 ± 7.59	79.64 ± 6.79	
BAD-D	C-CXL	5.40 ± 1.02	4.43 ± 1.04	4.33 ± 1.06	4.32 ± 1.13	4.30 ± 1.00	0.404 / 0.29
	A-CXL	5.08 ± 1.13	4.04 ± 1.22	4.17 ± 1.17	4.13 ± 1.27	4.07 ± 1.09	

5. Repeated-measures ANOVA/repeated-measures modeling

Repeated-measures modeling demonstrated significant temporal changes in tomographic, visual, and biomechanical parameters following CXL. A strong main effect of time was observed for DA, SP-A1, applanation times, PD, and HC radius (all p < 0.001), consistent with postoperative remodeling across protocols.

A main effect of group was not significant for the principal biomechanical endpoints (p > 0.05), and most time × group interaction terms were not significant, indicating broadly parallel improvement patterns across protocols. Importantly, a significant time × group interaction was detected for SP-A1 (p=0.042), supporting a modest protocol-dependent difference in stiffness trajectory over time (**Table 7**).

**Table 7:** Repeated-measures mixed-model (RM-ANOVA-style) fixed-effect tests (Wald χ^2^)

Measure	Effect	df	Wald_chi2	p	N_eyes	N_obs
Kmax	Group	1	0.1422	0.7060791675734436	63	315
Kmax	Time	4	251.2	3.666712071642264e-53	63	315
Kmax	Group: Time	4	3.36	0.4995541077056428	63	315
UCVA (logMAR)	Group	1	0.2157	0.6423495865072659	63	315
UCVA (logMAR)	Time	4	302.4	3.3091443951511354e-64	63	315
UCVA (logMAR)	Group: Time	4	2.499	0.6448004403813383	63	315
CDVA (logMAR)	Group	1	0.7687	0.3806154545233932	63	315
CDVA (logMAR)	Time	4	641.5	1.6534224922994408e-137	63	315
CDVA (logMAR)	Group: Time	4	2.499	0.644830181389594	63	315
Pachymetry (µm)	Group	1	1.52	0.21756687940616362	63	315
Pachymetry (µm)	Time	4	403	6.377831558693958e-86	63	315
Pachymetry (µm)	Group: Time	4	1.16	0.8845958062153152	63	315
Deformation Amplitude (mm)	Group	1	0.1412	0.7071221230911419	63	315
Deformation Amplitude (mm)	Time	4	393.6	6.849442034991932e-84	63	315
Deformation Amplitude (mm)	Group: Time	4	4.885	0.29927759853843194	63	315
SP-A1	Group	1	0.1605	0.6886728779434117	63	315
SP-A1	Time	4	349.7	2.0039596740612354e-74	63	315
SP-A1	Group: Time	4	9.89	0.04231767869652856	63	315
HC Radius (mm)	Group	1	0.1898	0.6630882442961582	63	315
HC Radius (mm)	Time	4	620.2	6.729255487391533e-133	63	315
HC Radius (mm)	Group: Time	4	2.316	0.6777862327882052	63	315
BAD-D	Group	1	1.288	0.2564101343088986	63	315
BAD-D	Time	4	345	2.1170630509371532e-73	63	315
BAD-D	Group: Time	4	8.021	0.0908197671226558	63	315

6. Correlation analysis

Correlation analysis demonstrated relationships between biomechanical reinforcement and clinical/tomographic outcomes, supporting a mechanical link between stromal stiffening and postoperative remodeling. Changes in stiffness-related parameters (particularly SP-A1) were associated with changes in corneal geometry and ectasia indices over follow-up, consistent with the concept that CXL effects are fundamentally biomechanical.

7. Long-Term stability and overall interpretation

Long-term follow-up demonstrated sustained corneal stabilization in both treatment groups, with no evidence of late regression. After the initial postoperative remodeling phase, biomechanical and tomographic parameters remained stable between 24 and 36 months. Kmax values showed no significant increase during the final year of follow-up in either group (p > 0.05), confirming persistent topographic flattening. Similarly, UCVA and CDVA gains achieved during the first two postoperative years were maintained at 36 months, with no eye losing ≥ 2 Snellen lines of corrected vision.

Biomechanical parameters exhibited a plateau pattern after 24 months. SP-A1 remained elevated, and deformation amplitude remained reduced, with no significant reversal of stiffening in either protocol. This suggests that the cross-link-induced stromal reinforcement is biologically stable over time. The highest concavity radius and applanation timing metrics also showed sustained changes, further supporting long-term mechanical integrity of the cornea.

Pachymetric values stabilized after the recovery phase, with no progressive thinning observed beyond 24 months. The conventional CXL group demonstrated a near return toward baseline thickness, while the accelerated group maintained slightly lower but stable values, indicating structural remodeling rather than ongoing tissue loss.

BAD-D index values continued to reflect ectasia stabilization, with no tomographic signs of renewed progression. Importantly, no case required retreatment or showed clinical progression during the third postoperative year.

Overall, the absence of biomechanical or tomographic regression indicates that both protocols provide durable disease stabilization, with the conventional protocol producing a higher long-term biomechanical set point. These findings support the concept that corneal cross-linking establishes a new equilibrium state of stromal rigidity that remains stable for at least three years post-treatment.

## Discussions

The present results confirm that both epi-off conventional and accelerated CXL achieved durable clinical and biomechanical stabilization over 36 months in progressive keratoconus. At the final follow-up, no significant between-group differences were observed for Kmax, visual acuity, pachymetry, deformation amplitude, applanation times, peak distance, HC radius, SP-A1, or BAD-D, indicating comparable overall efficacy at three years. Nevertheless, the significant time × group interaction for SP-A1 suggests that the kinetics of stromal stiffening may differ between protocols, with irradiation duration potentially influencing the time-course of biomechanical reinforcement even when total fluence is equivalent. These findings are consistent with prior comparative and longitudinal studies showing sustained stiffening after CXL and only modest protocol-related differences in the time-course of biomechanical recovery [15,22-26].

In this 63-eye cohort (34 conventional CXL; 29 accelerated CXL) followed for 36 months, both protocols effectively stabilized keratoconus, topography, and vision. Longitudinal analysis demonstrated significant biomechanical strengthening in both groups, reflected by a reduction in deformation amplitude (DA), an increase in SP-A1, delayed applanation times, and favorable changes in the highest concavity parameters. At the final 36-month time point, absolute inter-group differences were small and not statistically significant for most parameters; however, longitudinal modeling revealed a significant time × group interaction for SP-A1, indicating a modest protocol-dependent divergence in stiffness trajectory over time. The most pronounced biomechanical changes occurred between baseline and 12-24 months, consistent with progressive corneal remodeling after cross-linking that tends to plateau during the second postoperative year, as reported in multiple longitudinal studies [23-30].

**Fig. 15:**
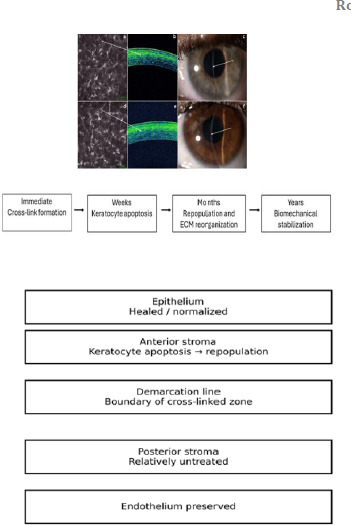
In vivo confocal microscopy (IVCM), anterior segment OCT, slit-lamp photography, and a biological timeline of corneal remodeling after CXL

From a mechanistic standpoint, this pattern aligns with the known physiopathology of keratoconus, in which stromal weakening results from microstructural disorganization, altered extracellular matrix turnover, and increased proteolytic and oxidative stress activity. Histopathologically, keratoconus corneas show epithelial thinning, Bowman’s layer fragmentation, stromal thinning with lamellar slippage, and keratocyte alterations, creating a structurally fragile load-bearing architecture [4-5]. These baseline abnormalities explain the heightened corneal deformability observed preoperatively and the clinical importance of interventions that restore mechanical resistance. Histopathological, epi-off CXL produces a characteristic tissue response. Early after treatment, anterior stromal keratocyte apoptosis is followed by repopulation and extracellular matrix reorganization. The stromal demarcation line represents the transition between cross-linked anterior stroma and untreated posterior stroma and is considered a surrogate marker of treatment depth [6]. Histologic examinations of corneas previously treated with CXL have demonstrated persistent structural changes compatible with long-term remodeling rather than transient effects [7]. These cellular and matrix-level changes provide biological support for the sustained biomechanical shifts detected by Corvis ST parameters in our cohort (**Fig. 15**).

The present 36-month data demonstrate sustained corneal stabilization after both epi-off protocols, with durable biomechanical reinforcement maintained between 24 and 36 months. Although final absolute values were comparable, the SP-A1 trajectory difference suggests that the irradiation profile may influence the kinetics of stiffening rather than simply its end-point magnitude. This mid-term biomechanical trajectory is consistent with the long-term Romanian experience. Nicula et al. reported at 7 years that standard epi-off CXL induced persistent Kmax reduction and stable UCVA/CDVA improvement without late progression, indicating durable stromal reinforcement beyond the early postoperative phase [27]. Their subsequent 10-year follow-up confirmed maintained keratometric flattening and visual stability, with no severe late complications, supporting the concept that cross-linking establishes a long-lasting biomechanical equilibrium [28]. Our 3-year findings likely represent the earlier portion of this same remodeling continuum.

International long-term cohorts show similar durability. O’Brart et al. demonstrated that at 7 years, keratometric flattening and visual improvements persisted, with no treated eyes progressing [29]. Greenstein et al. reported 10-year stability of visual acuity and topography in pivotal-study patients, reinforcing that epi-off CXL provides sustained disease control [30]. These reports align with our absence of late regression and stable biomechanics between 24 and 36 months.

Our study adds value by showing protocol-dependent biomechanical kinetics using Corvis ST. Felter et al. observed that severity indices may fluctuate in the first postoperative year, partly due to CXL-related thinning, while longer-term follow-up demonstrated stabilization; they emphasized that stiffness-linked parameters remain more reliable for tracking biomechanical recovery [22]. Sedaghat et al. similarly reported progressive biomechanical evolution over 4 years, correlating changes in stiffness with visual outcomes [23]. These observations support our use of SP-A1 and DA as primary efficacy markers and help interpret the early thinning followed by recovery seen in our pachymetry data.

Evidence regarding accelerated CXL suggests measurable, though sometimes smaller or slower, biomechanical effects. Xanthopoulou et al. confirmed that Corvis ST parameters remain reliable for assessing keratoconus corneas ≥ 2 years after accelerated CXL, validating long-term biomechanical monitoring [24]. Jian et al. reported increased stiffness at 1 year after accelerated transepithelial CXL in pediatric keratoconus, although longer follow-up was needed [25]. Together, these findings indicate that both protocols stabilize ectasia, while the irradiation time may influence the time course of mechanical consolidation rather than the ultimate clinical outcome.

Although the Corvis Biomechanical Index (CBI) and Tomographic Biomechanical Index (TBI) are valuable tools for detecting ectatic corneas, they are not designed to quantify treatment success after CXL. Both indices were developed as diagnostic classifiers, integrating deformation behavior, pachymetric distribution, and tomographic asymmetry to differentiate normal from ectatic corneas rather than to measure intrinsic stromal stiffness [31-35]. Following CXL, the cornea may become biomechanically stronger while still retaining geometric and structural characteristics of keratoconus, such as posterior elevation and regional thinning. Consequently, CBI and TBI values may remain elevated or change only modestly despite effective biomechanical reinforcement [34,35].

Furthermore, these indices incorporate pachymetry-dependent variables that are influenced by the early postoperative thinning phase, potentially altering index values independently of true mechanical improvement [36]. In contrast, parameters such as stiffness parameter at first applanation (SP-A1), deformation amplitude, and highest concavity radius directly reflect the cornea’s dynamic response to mechanical loading and therefore represent more appropriate markers of biomechanical efficacy after CXL [37]. For these reasons, CBI and TBI should be interpreted as screening and risk-detection tools rather than endpoints for assessing cross-linking success.

The clinical takeaway is that untreated keratoconic corneas typically demonstrate abnormal (ectasia-pattern) CBI and TBI values, reflecting altered biomechanical behavior and tomographic asymmetry with weakened biomechanical integrity; however, CBI/TBI as detection and risk indices are not ideal metrics of CXL success [31-35,38].

### Interpretation

The finding of somewhat greater biomechanical effect with C-CXL suggests that for eyes where maximal biomechanical reinforcement is a priority (for example, very progressive cases or advanced thinning), C-CXL remains a strong option. However, accelerated protocols still showed stabilization and improved biomechanics - and may be preferable for patient comfort and operating efficiency. A-CXL may produce shallower cross-link distribution, leading to reduced biomechanical reinforcement. Long-term corneal stability depends more on treatment duration than fluence equivalence. Importantly, the observed differences, while measurable on Corvis ST, were modest in magnitude and did not translate into large differences in K-max or visual acuity by 36 months.

In clinical practice, the choice of protocol should take into account patient factors, disease severity, and planning considerations.

## Conclusions

At three years, both epithelium-off conventional and accelerated corneal collagen cross-linking effectively stabilized keratoconus, confirming the long-term efficacy of CXL in halting disease progression. However, C-CXL produced greater biomechanical reinforcement, evidenced by a larger increase in SP-A1, greater reduction in deformation amplitude, and more pronounced changes in the highest concavity parameters.

Keratometric flattening correlated strongly with increased corneal stiffness, indicating that topographic improvement after CXL is mechanically driven. Visual acuity gains paralleled reductions in corneal deformability, supporting the link between biomechanical stabilization and optical performance.

Pachymetric changes followed the expected pattern of early thinning and subsequent stromal remodeling, with long-term stability in both groups and more complete recovery after conventional treatment.

Dynamic Scheimpflug imaging proved sensitive for detecting these biomechanical changes and for differentiating protocol-dependent effects. Parameters directly reflecting mechanical behavior, particularly SP-A1 and deformation amplitude, were the most informative markers of treatment efficacy.

Overall, while both protocols achieved disease stabilization, conventional epi-off CXL provided stronger long-term biomechanical strengthening, suggesting that irradiation time remains a relevant factor in determining the depth and durability of stromal cross-linking.

**The c**linical implication of the study is that keratometric flattening was not random but **mechanically driven**. Why this is powerful - corneal tomography alone cannot prove biomechanics. This correlation showed that b**iomechanical strengthening leads to anatomical flattening**.

This study supports CORVIS ST as a **true efficacy biomarker**, not just a monitoring device.

### Limitations

This 3-year cohort provides valuable mid-term biomechanical data, but several limitations warrant consideration. The sample size, with unequal protocol distribution, reduces power for detecting subtle inter-protocol differences and may inflate effect size estimates. Inclusion of both eyes from some patients introduces inter-eye correlation that, while partly addressed with repeated-measures analysis, would be better modeled using mixed-effects approaches.

The study was prospective but non-randomized and unmasked, which may have introduced selection bias and confounding by disease phenotype, cone location, atopy, and progression behavior. As a single-center study, outcomes may reflect device- and protocol-specific factors, limiting generalizability to alternative CXL platforms or modified accelerated techniques.

Corvis ST metrics are influenced by IOP, thickness, and corneal geometry; early postoperative thinning and hydration changes may independently alter deformation indices, independent of intrinsic stromal stiffening. Moreover, the device measures global deformation and may not fully capture localized biomechanics of the cone.

Functional visual quality metrics and patient-reported outcomes were not assessed, and the very early postoperative phase was not evaluated. While 36 months represents meaningful mid-term follow-up, longer observation is required to confirm durability and detect rare late events.

Accordingly, although the data suggest greater biomechanical reinforcement after conventional epithelium-off CXL, protocol-dependent differences should be interpreted cautiously and validated in larger, randomized, multi-center studies with advanced statistical modeling and broader functional endpoints.
